# Inequality in Addiction and Mental Disorders in 6818 Suicide Attempts: Determine of Positive Contribution of Determinants by Decomposition Method

**Published:** 2017-06

**Authors:** Yousef VEISANI, Ali DELPISHEH, Ghobad MORADI, Jafar HASSANZADEH, Kourosh SAYEHMIRI

**Affiliations:** 1. Psychosocial Injuries Research Center, Ilam University of Medical Sciences, Ilam, Iran; 2. Dept. of Clinical Epidemiology, School of Health, Ilam University of Medical Sciences, Ilam, Iran; 3. Social Determinants of Health Research Center, Kurdistan University of Medical Sciences, Sanandaj, Iran; 4. Research Center for Health Sciences, Dept. of Epidemiology, School of Health, Shiraz University of Medical Sciences, Shiraz, Iran; 5. Dept. of Biostatistics, School of Health, Ilam University of Medical Sciences, Ilam, Iran

**Keywords:** Inequality, Addiction, Decomposition, Suicide attempts, Mental disorders

## Abstract

**Background:**

Unpleasant health outcomes are common in deprived communities and have been shown a direct connection between socioeconomic status and wellbeing but, the effect of the socioeconomic status on suicide is currently controversial. This study explored the relationship of the socioeconomic status and addiction and mental disorders in suicide attempts and decomposed inequalities into its determinants to calculating share of contribution to the inequality.

**Methods:**

This cross-sectional study recognized 546 suicide deaths and 6818 attempted suicides from 1 Jan 2010 to 31 Dec 2014 in Ilam Province, West of Iran. Inequality measured by concentration index (CI) and decomposing contribution in inequality. All analysis was performed by standard statistical software, Stata (Ver. 11.2).

**Results:**

The pooled rate of death in people with suicide attempts was 8.0% and that of having a history of suicide was 5.2%. The overall CI for addiction was −0.233 (95% CI= −0.383 to −0.084), and the CI for mental disorders was 0.0006 (95% CI = −0.009 to 0.010). The more amount of contribution to socioeconomic inequality in suicide attempts because of age group of < 25 yr (26%), socioeconomic level (23%), and marital status (22%).

**Conclusion:**

Addiction and mental disorders were distributed among suicide attempters unequally and gap between advantaged and disadvantaged attempted suicide confirmed by our results. Addiction prevention-related policies and programmers’ should be focus on disadvantaged groups.

## Introduction

Suicide ranks among the top 10 causes of death, and it is the second-leading cause of death for the 15–19 age groups in many Western countries (1). Eighty-four percent of the new suicide cases will arise in low and middle-income developing countries (2). In globally the prevalence rates of parasuicide in lifetime was reported 720 to 5.930 per 100 (3). In 2014, systematic review of the Eastern Mediterranean Region reported that the incidence of committed suicide ranged from 0.55 to 5.4 per 100000 (4). Death from an attempted suicide is 100 times greater than the mortality rate in the general population (5). According to the existing literature, the most important risk factors for suicide include being young, being female, being unemployed, being single or divorced, having a mental disorder, and having a previous history of suicide attempts (6).

Psychiatric disorders are the most common determinants in suicide attempts (7). Increased risk of suicide resulting in major depression, bipolar disorder, schizophrenia has been shown (8). In Iran, type of psychiatric disorders was an associate factor to attempted suicide (9). Other study showed % 45.3 of the attempters has at least one psychiatric disorder during lifetime, on the other hands 22% of them suffering from major depressive disorder. The literature review shows 26–55 age years old, married people, highly educated persons, females, retired and lived in the urban areas were the most related factors to suicide attempts in Iran that similar to those in Western countries (10). Addiction in survey reports was an important factor to suicide attempts in Iran (11).

Unpleasant health outcomes are common in deprived communities, and a direct connection has been shown to exist between socioeconomic status and wellbeing (12); however, the effect of socioeconomic status on suicide is currently controversial. A lower economic status increases the risk of suicide attempts (13, 14), while other results oppose this finding (15). These conflicting results could be due to varying methods being used to measure socioeconomic status.

Calculating socioeconomic inequality in health outcomes has been common around the world (particularly in developed countries). In order to reduce socioeconomic inequalities in the coming decades we should be enabling to more knowledge about it. The Concentration Index (CI) is efficient measure to calculating of inequalities and specifies the positive contributions determinants in inequality.

This study was conducted to identify the relationship between addiction and mental disorders’ inequality in suicide attempts using an inequality scale and a decomposition approach.

## Materials and Methods

### Study subjects

Data of suicide attempts were extracted from the systematic registration suicide data (SRSD) system provided by Ilam University of Medical Sciences, Ilam Province, Iran. Data on attempted suicide have been collected systematically since 2010.

This study recognized 546 suicide deaths and 6272 attempted suicides from 1 Jan 2010 to 31 Dec 2014. The data for the present study consisted of all suicide attempts in the Ilam Province of Iran by residents’ ages six years and older admitted to healthcare sections during the period of study. In systematic registration suicide data (SRSD), the inclusion criterion was all registered cases of suicide attempt 2010 to end of 2014 that recorded in SRSD.

We exclude cases with lack of reliable data such as socioeconomic status variables. Suicide attempts were determined through the analysis of physician claims and hospital admission records, in addition to daily suicide counts according to a structured schedule, which involved nine items: age, sex, marital status, educational level, job status, partners’ job status and educational level, region of residence, race, and other demographic information. Data concerning mental disorders, addiction, methods of suicide attempt, and outcome were collected from individual outpatient visits on a monthly basis.

### Definition of Variables

Outcome variable was suicide deaths and attempted suicides, independent variables included: age at the time of suicide (6–15 ; 16–25 ; 26–35 ; 36–45 ; 46–55 ; 56–65, and >65 yr old), attempters gender, the educational level (five levels: illiterate, primary school, high school, diploma, university), location of residence (urban/rural), job status, having a history of attempted suicide, addiction (alcohol addiction, substance abuse, etc.), and had a mental disorders (mental disorders include depression, anxiety disorders, schizophrenia and eating disorders), socioeconomic status (We calculated the SES index by applying principal component analysis (PCA) to the eight SES constituent items: sex, educational level of suicide attempters, partners educational level, marital status, job status, partners job, location of residence, and financial status). In decomposition procedure, we defined dummy variables, low socioeconomic status is that including socioeconomic in the bottom 40% was created to determine rate of participation variables to inequality. In the following binary variable illiterate and primary school were grouped to decomposition analysis, age years group of <25, and unemployed (homemaker not included) were other binary variables in decomposition analysis.

### Statistical analyses

SES was calculated using PCA procedure in order to identify variables with greater impact overall variance. Using this Procedure, new variables that represent SES is identified (16). Initially, we created dummy (0/1) for nominal variables such as residence and job status and 8 variables enrolled in PCA.

Inequality is calculated by measuring the concentration index (CI). According to the Wag staff article (17), most researchers have used this index to measure inequality. The CI is the cumulative percentage of variables against the cumulative percentage of population, ranked by economic index from poorest to richest. If there is no inequality in the distribution, CI is zero. When the curve is above of equality line CI is negative and variable is concentrated in those with low SES and positive CI reminders more concentration of variable in those with high-grade SES. By the mathematical formula, CI can be computed as twice the covariance of the health variable and a person’s rank in terms of economic status, divided by the mean of the health variable $C=\frac{2}{\mu }\text{cov}(\text{YiRi})$ where yi and Ri are respectively the health status of the ith individual and the fractional rank of the ith individual (in terms of the index of household economic status); μ is the mean of the health and Cov denotes the covariance (18). Decomposition proposed to determine contribution of socioeconomic covariates to inequality. This approach allows one to determine rate of participation variables to inequality (19). We used to decomposition approach for the decomposing socioeconomic determinants analysis, quintiles 1 and 2 and quintiles 3, 4, and 5 were grouped together. This created a binary low SES variable that including socioeconomic in the bottom 40% of this approach allows one to determine rate of participation variables to inequality. For this approach we denote total score of SES by y and the set of covariates by X=x_1_, x_2_,…, x_k_ linear model using an approach, y=α+∑ βkXk + ∈ were, α and ∈ denote respectively, constant and error term. In a next step we can obtain the contribution of each determinant to inequality by multiplying the elasticity of each determinant by its concentration index(βkXk/μ)Ck where μ is the mean of y and CIs for determinants (Ck). This is the absolute contribution of each determinant to the measured inequality. In final step we calculate percentage contribution of each determinant simply through dividing its absolute contribution by the concentration index of the health variable $\left(\frac{\beta \text{kXk}}{\mu }\right){\text{Ck}}_{\bar{c}}$ (20). We used standard statistical software, STATA (Ver. 11.2).

## Results

The study population’s characteristics are shown in Table 1. The proportion of females was 53.7%, while 58.5% of the study sample consisted of people between 16–25 yr of age, 56.9% were single, 76.1% lived in a rural area, 32.6% were housewives, and 40% had a college degree.

**Table 1:** Successfully committed suicide and having history of suicide in terms of determinant variables among suicide attempters

**Characteristics of Persons Attempted suicide**	**No. of suicide (%)**	**Successfully committed suicide**	**Having history of suicide**
**Age group (yr)**			
6–15	156 (2.3)	9(5.8)	9 (5.8)
16 – 25	3782 (55.5)	229 (6.1)	186 (4.9)
26–35	2185 (32.0)	186 (8.5)	132 (6.0)
36–45	415 (6.1)	49 (11.8)	14 (3.4)
46–55	178 (2.6)	45 (25.3)	10(5.6)
56–65	70(1.0)	14 (20.0)	2 (2.9)
> 66	32 (0.5)	14(43.8)	1 (3.1)
**Sex**			
Male	3157 (46.3)	225 (8.1)	183 (5.8)
Female	3661 (53.7)	291 (7.9)	171 (5.2)
**Residence**			
City	1631 (23.9)	176 (10.8)	78(4.8)
Village	5187 (76.1)	370 (7.1)	276 (5.3)
**Job**			
Unemployment	2143 (31.4)	186 (8.7)	133 (6.2)
Housewife	2220 (32.6)	190 (8.6)	119 (5.4)
Student	504 (7.4)	42 (6.7)	36 (7.9)
Employee	139 (2.0)	20 (14.4)	5 (3.6)
Free Job	771 (11.3)	60 (8.0)	50 (6.5)
Other	1138 (16.7)	77 (6.0)	11 (.009)
**Educational level**			
Illiterate	773 (11.3)	133 (17.2)	35 (4.5)
Primary school	401 (5.9)	82 (20.4)	20 (5.0)
Guidance/high school	1988 (29.2)	157 (7.9)	129 (6.5)
Diploma	2775 (40.7)	136 (4.9)	139 (5.0)
University	881 (12.9)	38 (4.3)	31 (3.5)
**Marital statues**			
Marriage	2937 (43.1)	289 (9.8)	161 (5.5)
Single	3881 (56.9)	257 (6.6)	193 (5.0)
**Total**	6818(100)	546(8.0)	354 (5.2)

The prevalence of death from suicide attempts was 8.1% for males and 7.9% for females (*P*=0.440), while 5.8% of males and 5.2% of females had a history of suicide attempts (*P*=0.021). The pooled rate of death in people with suicide attempts was 8.0% and that of having a history of suicide was 5.2%. A significant relationship was shown between all of the grouped characteristics, death, and a history of suicide *(P*<0.001).

**Fig. 1:**
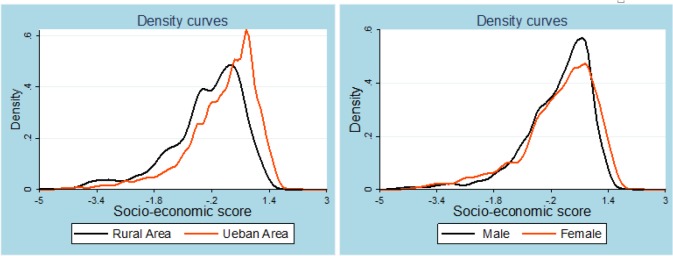
Distribution of socio-economic scores

This study’s results show evidence for an internal coherence of socioeconomic scores for urban and rural and for male and female suicide attempts. The density curves of the socioeconomic scores for rural and urban settings and for males and females are shown in Fig. 1.

The distribution of scores tends to follow a normal curve for rural and urban areas, but it skews to the left for males and females.

**Fig. 2:**
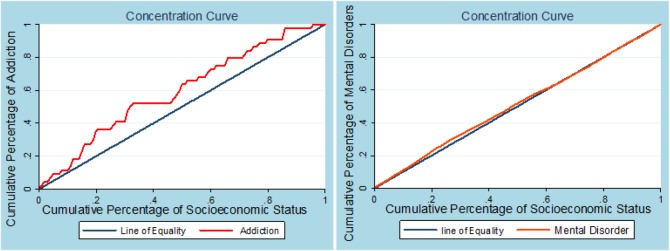
Concentration curve of addiction and mental disorders in persons attempted suicide

**Fig. 3:**
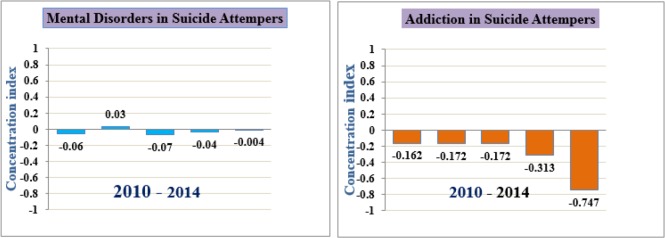
Trends in the concentration indices of addiction and mental disorders among suicide attempters Ilam province (2010–2014)

The concentration curve is presented in Fig. 2, which shows that suicide attempters with lower socioeconomic status have a greater risk for addiction. The overall CI for addiction was −0.233 (95% CI = −0.383 to −0.084), and the CI for mental disorders was 0.0006 (95% CI = −0.009 to 0.010). Fig. 3 shows trends in the concentration indices of addiction and mental disorders among suicide attempters in the Ilam Province from 2010 onward. Disparities existed in the prevalence of addiction and mental disorders across socioeconomic groups. More inequality was observed in addiction compared to mental disorders, but the trend showed an inverse positive slope in both cases.

Through the decomposition method, we created a dummy variable for all the variables with several categories and presented the information in Table 2 that shows the more amount of contribution to socioeconomic inequality in suicide attempts because of age group of < 25 yr (26%), socioeconomic level (23%), and marital status (22%) and 6% was the residual component, which means it could not be explained by the data entered into the model.

**Table 2:** Decomposition analysis of socio-economic determinants of successfully committed suicide

**Determinants**	**Coefficient**	***t***	***P*-value>|*t*|**	**Absolute Contribution**	**Relative Contribution (%)**
Sex (Female)	.164	8.39	<0.001	0.004	0.04
Residence (rural areas)	.175	8.23	<0.001	0.003	0.04
Marital status (married)	.525	23.48	<0.001	0.025	0.22
Educational level (Illiterate and primary school)	.515	17.31	<0.001	0.014	0.12
Household economic status (Poorest and second poor)	−0.530	1.39	<0.001	0.026	0.23
Age group (<25 yr)	−.530	−27.8	<0.001	0.030	0.26
Job (unemployed)	.031	2.57	0.165	0.000	0.01
Partners educational level (Illiterate and primary school)	.076	2.57	0.010	0.001	0.01
Partners Job (unemployed)	−.030	−1.18	0.239	0.000	0.01
Cons	−0.155	−1.43	0.154	0.000	0.00
Residual				0.007	0.06
Total				0.115	1.000

* Elasticity indicates the impact of each determinant on the death from suicide attempts. How much change in the dependent variable is associated with one unit of change in the explanatory variable?

## Discussion

This study used the Ilam Province’s systematic registration suicide data (SRSD) to determine the inequality in addiction and mental disorders between suicide attempters. We showed that addiction is not equal in all socioeconomic status groups of the population and that the distribution of addiction in suicide attempters with lower socioeconomic status is more common than in suicide attempters with a higher socioeconomic status. These findings suggest that struggling with addiction in low socioeconomic groups must be targeted at all age groups. Addiction is a risk for suicide attempts (21), since the rate of addictive behaviors is more common in deprived people; therefore, concentrating prevention programs on these people can potentially reduce suicide inequality. Similar results have been shown for perceived lower socioeconomic status, smoking, and suicide. We found that, in suicide attempters, the prevalence of mental disorders increased as the disadvantage increased. Furthermore, the mortality rate in subjects suffering from mental disorders was higher than in those that did not, 7.4% vs. 6.4%. Therefore, our results are similar to studies that show an association between deprivation and mortality in those with mental disorders in the general population. We found a significantly higher proportion of suicide in those with mental disorders in deprived quintiles. In one Australian study, a negative correlation between suicide and socioeconomic status has been found (22), and the same holds true for the US (23).

There are limited studies investigating the association between socioeconomic status and mental disorders in individuals with suicide attempts. However, there are studies that investigate the impact of socioeconomic status on suicide (24), par suicide (25), anxiety disorder (26), and bipolar disorder (27). The association between some socioeconomic determinants and suicide has been investigated in Iran. Married attempters, females, educated people, retired persons, and 26–55 age groups had more suicide attempts, as well, among attempters, 45.3% of them have had at least one psychiatric disorder, and major depressive disorder was reported in 22% of them (10).

Policy makers can use the decomposition method for planning and policy purposes. In this study, addiction is responsible for the highest positive contribution to inequality. However, addiction is more prevalent in the 25-or-less age group; thus, policymakers should pursue programs to enhance social welfare for this age group to decrease the positive contribution of addiction in the socioeconomic inequality of suicide attempters.

There are several limitations mentioned. First, due to the cross-sectional nature of this study, the inference of the significant relationship between socioeconomic status and suicide attempts is not conclusive. Second, this study’s data was from self-reported data, and self-reports have been suggested to contain a risk for bias. Third, we are limited to straight data, such as income, to measure socioeconomic scores.

## Conclusion

Addiction and mental disorders were distributed among suicide attempters unequally, and the gap between the advantaged and the disadvantaged suicide attempters. Our results suggest that addiction prevention-related policies and programs should focus on disadvantaged groups.

## Ethical considerations

Ethical issues (Including plagiarism, informed consent, misconduct, data fabrication and/or falsification, double publication and/or submission, redundancy, etc.) have been completely observed by the authors.
